# Mapping white matter structural covariance connectivity for single subject using wavelet transform with T1-weighted anatomical brain MRI

**DOI:** 10.3389/fnins.2022.1038514

**Published:** 2022-11-23

**Authors:** Xun-Heng Wang, Bohan Zhao, Lihua Li

**Affiliations:** Institute of Biomedical Engineering and Instrumentation, Hangzhou Dianzi University, Hangzhou, China

**Keywords:** structural covariance connectivity, white matter, wavelet transform, support vector regression, predictive models

## Abstract

**Introduction:**

Current studies of structural covariance networks were focused on the gray matter in the human brain. The structural covariance connectivity in the white matter remains largely unexplored. This paper aimed to build novel metrics that can infer white matter structural covariance connectivity, and to explore the predictive power of the proposed features.

**Methods:**

To this end, a cohort of 315 adult subjects with the anatomical brain MRI datasets were obtained from the publicly available Dallas Lifespan Brain Study (DLBS) project. The 3D wavelet transform was applied on the individual voxel-based morphology (VBM) volume to obtain the white matter structural covariance connectivity. The predictive models for cognitive functions were built using support vector regression (SVR).

**Results:**

The predictive models exhibited comparable performance with previous studies. The novel features successfully predicted the individual ability of digit comparison (DC) (*r* = 0.41 ± 0.01, *p* < 0.01) and digit symbol (DSYM) (*r* = 0.5 ± 0.01, *p* < 0.01). The sensorimotor-related white matter system exhibited as the most predictive network node. Furthermore, the node strengths of sensorimotor mode were significantly correlated to cognitive scores.

**Discussion:**

The results suggested that the white matter structural covariance connectivity was informative and had potential for predictive tasks of brain-behavior research.

## Introduction

Structural covariance connectivity, also named morphological connectivity or morphological similarity, has drawn significant research interests in neuroscience recently (Bethlehem et al., 2017; DuPre and Spreng, 2017; Wang et al., 2018b,^2020^; Duan et al., 2020; He et al., 2021; Xu et al., 2021). Compared to group-wise structural covariance network, the individual structural covariance connectivity exhibited diagnostic and predictive powers for personalized evaluation of brain disorders and developments (Wang et al., 2018a,^2020^; Gao et al., 2020; He et al., 2021; Li et al., 2021). Furthermore, the structural covariance connectivity could reflect the gene-expression in the human brain (Seidlitz et al., 2018). However, current studies of structural covariance connectivity were focused on gray matter using brain morphological features, the white matter morphological connectivity remains largely unexplored. The topology of white matter was always investigated using diffusion tensor imaging (Wang et al., 2012). There were quite a few studies that probe white matter connectivity using alternative MRI protocols (i.e., T1-weighted MRI, T2-weighted fMRI). Given recent progresses in constructions of white matter functional connectivity using BOLD fMRI (Gore et al., 2019; Li et al., 2019; Huang et al., 2020; Wang et al., 2021), the white matter morphological connectivity based on structural MRI might also be informative.

The structural covariance connectivity estimators were always based on feature similarity of morphological measures (Wang et al., 2020; Li et al., 2021). The key procedure to construct structural covariance connectivity for single subject was deriving significant regional morphological features from T1-weighted brain images. Surface-based morphology (SBM) can provide vertex-wise morphological measures (i.e., thickness, volume, area, folding, and curvature) as feature vectors. However, the classical voxel-based morphology (VBM) can only produce a volume value for each voxel. To obtain voxel-wise morphological feature vectors is a challenging task. Radiomic analysis considered the medical imaging as digital data, which can yield a significant number of features (Gillies et al., 2016; Lambin et al., 2017). Radiomic features have been applied in diagnostic models for brain disorders (Lui et al., 2016; Sun et al., 2018). To our knowledge, conventional radiomic features were regional measures rather than interregional measures. Recently, we proposed a wavelet-based method to extract voxel-wise structural covariance networks (Wang et al., 2018b). The wavelet features that were an important component of radiomic measures contained both local and global brain structural attributes, which were beneficial for constructing brain networks (Hackmack et al., 2012; Canales-Rodriguez et al., 2013; Wang et al., 2018b). So far, the validity of the white matter structural covariance connectivity based on the wavelet transform remain unexplored.

This paper aimed to map the white matter structural covariance connectivity from individual anatomical MRI, and to build predictive models for cognitive functions based on the interregional features. In the section “Materials and methods,” a group of 315 subjects were obtained from the Dallas Lifespan Brain Study (DLBS) project. The anatomical MRI datasets were preprocessed using the standard procedure of VBM. Then, wavelet transform was applied to the VBM dataset to obtain regional feature vectors. The white matter structural covariance connectivity was computed based on the regional wavelet features. The predictive models for cognitive functions were solved using feature selection and support vector regression (SVR). In the section “Results,” the performance of the predictive models and the predictive patterns were reported using machine learning. The relationships between the white matter structural covariance and conventional VBM features were also compared. In the section “Discussion,” the performance of the machine learning models and the decoded predictive connectivity patterns were discussed with previous evidences. The biological meanings of the proposed metrics were also discussed. Finally, we made the conclusions that the white matter structural covariance connectivity was informative and had predictive powers for brain-behavior tasks.

## Materials and methods

### Participants and MRI protocols

A cohort of 315 adult subjects were obtained from the DLBS project, which aimed to investigate the brain cognitive function across adult life span. The DLBS was a publicly available dataset which can be used for academical research with the creative commons license. For each subject, an anatomical MRI dataset was collected with a scan resolution of 256 × 256 × 160, field of view = 204 mm × 256 mm × 160 mm. In addition, the cognitive functions were investigated using different cognitive tasks. An amyloid PET volume as well as the APOE gene information were also obtained for a subgroup of the participants. All of the 315 subjects were included in this study. There were 117 male subjects (age = 54.49 ± 20.45) and 198 female subjects (age = 54.69 ± 19.93). The cognitive functions for each participant were evaluated using several clinical scales [i.e., Cambridge neuropsychological test automated battery (CANTAB), letter number sequencing (LNS)]. The detailed information (MRI parameters, demographical information, as well as cognitive scores) for this dataset could be found at the DLBS website.^1^

### Classical gray matter voxel-based morphology

The raw anatomical MRI datasets were preprocessed using the standard procedure of VBM, which was carried out using the FSL package (Good et al., 2001).^2^ First, the raw MRI images were brain-extracted using fslvbm_1_bet script and segmented to obtain the gray matter, white matter as well as cerebrospinal fluid. Then, the skull-stripped images were non-linearly registered to the Montreal Neurological Institute (MNI). standard brain space using FSL’s FNIRT algorithm. Third, the normalized images were averaged and flipped between left and right to construct a study-specific brain template using fslvbm_2_template script. Fourth, the raw gray matter images as well as white matter images were normalized to this template using the spatial transformation parameters. Finally, the normalized gray matter images were multiplied by the Jacobian of the non-linear deformation field to obtain the modulated images as the gray matter VBM features using fslvbm_3_proc script. The modulated images were smoothed using a Gaussian kernel with sigma = 3 mm. For each voxel, the gray matter VBM value indicates the voxel-wise volume of the location (i.e., the coordinates in x–y–z space). In addition, the white mater VBM features were extracted using the same pipelines.

### Whiter matter structural covariance connectivity

**FIGURE 1:**
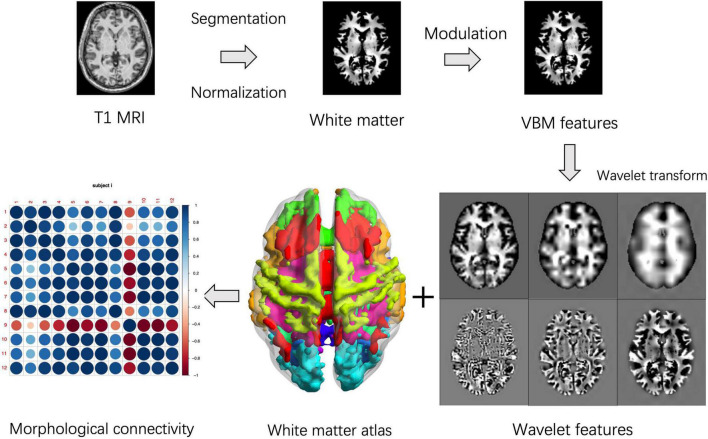
Pipelines for white matter structural covariance connectivity extraction. For each subject, the voxel-based morphology (VBM) transform is performed on the spatially normalized and modulated T-1 weighted white matter images. Then, 3D wavelet transform is performed on the individual VBM volume. Third, the white matter is assigned to 12 white matter networks (WMNs). Finally, the white matter structural covariance connectivity is computed based on correlation coefficients of the regional wavelet features. The VBM features, wavelets features and network features are significantly different from one of our previous study (Wang et al., 2018b).

One of our previous study applied wavelet transform to obtain voxel-wise brain morphological networks (Wang et al., 2018b). Here, we extended the aforementioned inter-voxel features to the interregional measures. The white matter structural covariance connectivity were computed using the following steps: (1) obtain the VBM features of the white matters using the same preprocessing steps in the section “Classical gray matter voxel-based morphology”; (2) apply 3D wavelet transform on the individual VBM dataset according to a previous study (Wang et al., 2018b); (3) get the 4D volume of wavelet features; (4) segment the individual wavelet-based volume into 12 white matter networks (WMNs) using a predefined atlas based on BOLD signal spatial clustering (Peer et al., 2017); (5) extract the mean wavelet features in each WMN to obtain the regional feature vector; (6) calculate the Pearson correlation coefficients of two wavelet feature vectors between each pair of WMNs; (7) obtain all Pearson correlation coefficients across WMNs as individual morphological connectivity matrix. Here, the level-three decomposition with near symmetric wavelet basis was applied in the 3D discrete wavelet transform based on Matlab’s wavedec3 function, since the connectivity features were reliable across different levels of wavelet decomposition according to our previous study (Wang et al., 2018b). The 3D volumes of wavelet decompositions were concatenated together to obtain the 4D volume of wavelet features. Figure 1 shows the pipeline of feature extraction. The detailed information of wavelet-based VBM transform could be found in a public available package.^3^ The structural covariance connectivity in the gray matter were extracted using a predefined brain atlas of 17 resting state networks (RSNs) for comparisons (Yeo et al., 2011). In addition, the proposed wavelet-based metrics were compared with the famous KL-divergence similarity (Kong et al., 2014).

### Predictive models using machine learning

The predictive models were solved using SVR based on the morphological connectivity. Since none of the nine cognitive variables was significantly correlated to age, the following equation was used in the predictive models:


$$C\,o\,g\,n\,i\,t\,i\,v\,e\,S\,c\,o\,r\,e=\sum_{i=1}^{66}w_{i}\,m\,c_{i}$$

In this equation, the *cognitive score* means the cognitive score predicted by machine learning. The *mc* means the interregional white matter morphological connectivity. The *w*_*i*_ is the weight of the morphological connectivity. There were 66 interregional features among the 12 WMNs. The indices and names of the brain networks could be found in a previous study (Peer et al., 2017). In this study, the cognitive scores included the Stockings of Cambridge (SOC), the stop signal task (SST), the spatial working memory (SWM), the verbal recognition memory (VRM), the digit comparison (DC), the digit symbol (DSYM), the ETS advanced vocabulary (ETSV), the ETS letter sets (ETSLS), and LNS.

In order to test the predictive power of the white matter morphological connectivity, the predictive models were trained and tested using 1,000 simulations of 10-fold cross-validations. In each fold of cross-validation, only features with significant correlations to cognitive scores (*p* < 0.05) were selected as inputs for the SVR training procedure. In each training fold, the default parameters of SVR were used to build predictive models with the e1071 package.^4^ However, the features in each training models were different from each other according to the feature selection procedure. Therefore, all of the testing models were based on the same SVR parameters but with different features. The onefold validation set is independent of the ninefolds feature selection and training processes. The performance of the predictive model was evaluated by the correlation coefficients with *p*-values, which represented the predicting accuracy between the original cognitive scores and the predicted scores. The final predictive patterns for cognitive functions were discovered by performing feature selection on the white matter morphological connectivity of all subjects measured with cognitive scales.

### Correlation analysis

In order to investigate the biological meanings of the proposed interregional metrics, correlation analysis was performed between the node strengths of the white matter morphological connectivity and the cognitive scores. The weighted brain network was built based on the significantly positive correlation coefficients (*r* > 0, *p* < 0.05) of the morphological connectivity. Positive and significant correlations were always used to build brain networks. Negative and non-significant correlations might be helpful for predictive models but were hard to explain the node attributes. The node strength was computed by the sum of connections related to the brain region. The procedure of node strength computation was implemented by the Brain Connectivity Toolbox.^5^

### Split-half analysis

For each predictive model, the subjects were reassigned into two groups: younger group (age < mean age) and elder group (age > mean age). There were 158 subjects in the younger group (age = 37.29 ± 10.59), and 157 subjects in the elder group (age = 72.06 ± 9.45). We first trained the nine predictive models based on the younger group and tested the models using the elder group. We then reversed the training and testing samples, and repeated the predicting procedure to investigate the age effects.

## Results

### Performance of the predictive models

**FIGURE 2:**
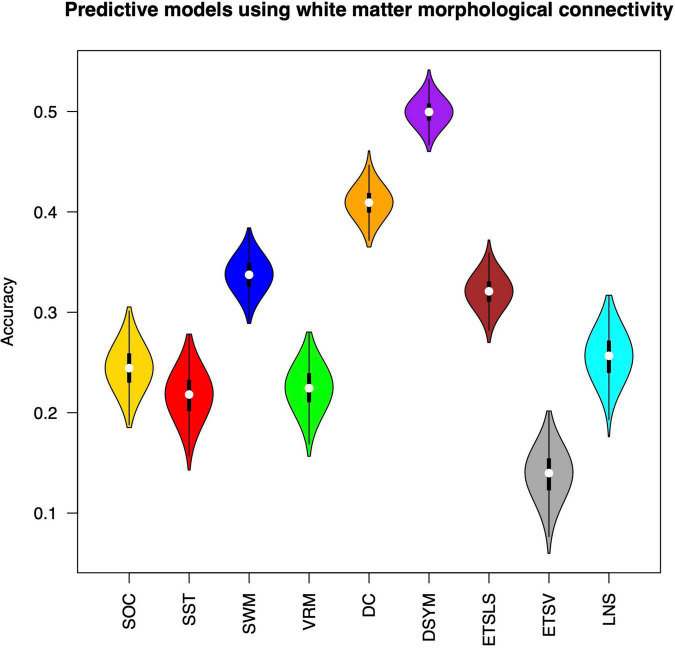
Performance of the predictive models based on white matter morphological connectivity. The nine cognitive predictive models include the Stockings of Cambridge (SOC), the stop signal task (SST), the spatial working memory (SWM), the verbal recognition memory (VRM), the digit comparison (DC), the digit symbol (DSYM), the ETS advanced vocabulary (ETSV), the ETS letter sets (ETSLS), and letter number sequencing (LNS).

Figure 2 shows the performance of the predictive models based on white matter structural covariance connectivity. The accuracy was indicated by the correlation coefficients between predicted values and original scores. Nine predictive models for cognitive scores estimations are established using 1,000 times of cross-validations. Most of the predictive scores are significantly correlated to original scores (*p* < 0.01). Table 1 shows the performance of the white matter predictive models. The individual abilities of DC [*r* = 0.41 ± 0.01, *p* < 0.01, mean absolute error (MAE) = 10.44 ± 0.11] and DSYM (*r* = 0.5 ± 0.01, *p* < 0.01, MAE = 9.73 ± 0.11) are successfully predicted using the novel features. The SWM can also be predicted (*r* = 0.34 ± 0.02, *p* < 0.01, MAE = 18.16 ± 0.21). The predictive models for ETSV exhibit the lowest performance. However, the ETSLS can be estimated using the structural covariance connectivity in the white matter (*r* = 0.32 ± 0.02, *p* < 0.01, MAE = 4.81 ± 0.05). The *p*-values for the performance of the predictive were not corrected by false discovery rate (FDR), since the *p*-values were computed through 1,000 times of permutations using the RVAideMemoire package.^6^

**TABLE 1:** Performance of white matter models.

Tasks	*r*	*P-value*	MAE
SOC	0.24 ± 0.02	*p* < 0.01	1.58 ± 0.02
SST	0.22 ± 0.02	*p* < 0.01	41.43 ± 0.43
SWM	0.34 ± 0.02	*p* < 0.01	18.16 ± 0.21
VRM	0.22 ± 0.02	*p* < 0.01	1.63 ± 0.02
DC	0.41 ± 0.01	*p* < 0.01	10.44 ± 0.11
DSYM	0.5 ± 0.01	*p* < 0.01	9.73 ± 0.11
ETSLS	0.32 ± 0.02	*p* < 0.01	4.81 ± 0.05
ETSV	0.14 ± 0.02	–	5.8 ± 0.06
LNS	0.26 ± 0.02	*p* < 0.01	2.3 ± 0.03

**FIGURE 3:**
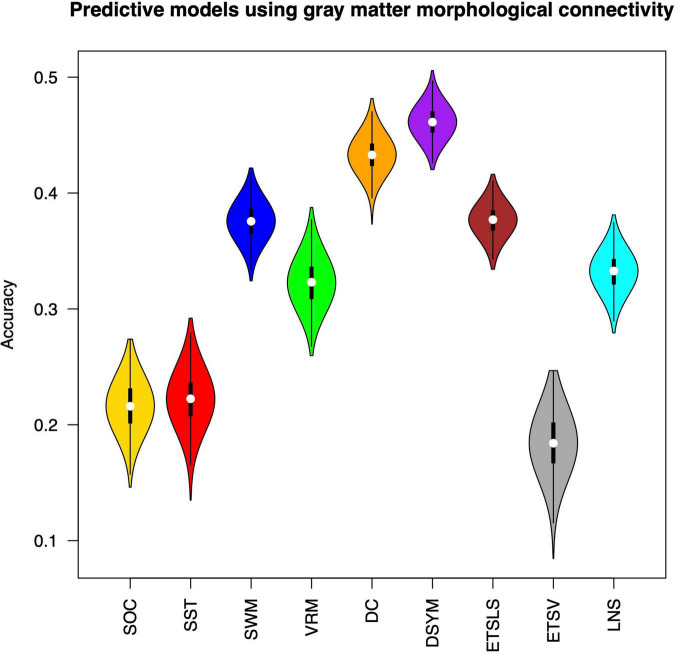
Performance of the predictive models based on gray matter morphological connectivity. The nine cognitive predictive models contain the Stockings of Cambridge (SOC), the stop signal task (SST), the spatial working memory (SWM), the verbal recognition memory (VRM), the digit comparison (DC), the digit symbol (DSYM), the ETS advanced vocabulary (ETSV), the ETS letter sets (ETSLS), and letter number sequencing (LNS), respectively.

Figure 3 shows the performance of the predictive models based on gray matter structural covariance connectivity. Nine predictive models for cognitive scores estimations are also established using 1,000 times of cross-validations. Most of the predictive values are significantly correlated to original scores (*p* < 0.01). Table 2 shows the performance of the gray matter predictive models. Similar to the predictive models based on white matter, the individual abilities of DC (*r* = 0.43 ± 0.01, *p* < 0.01, MAE = 10.31 ± 0.12) and DSYM (*r* = 0.46 ± 0.01, *p* < 0.01, MAE = 10.04 ± 0.11) are successfully predicted using the interregional gray matter features. The SWM can also be predicted (*r* = 0.38 ± 0.02, *p* < 0.01, MAE = 16.96 ± 0.19). The predictive models for ETSV exhibit the lowest performance. However, the ETSLS can be estimated using the structural covariance connectivity in the gray matter (*r* = 0.38 ± 0.01, *p* < 0.01, MAE = 4.63 ± 0.05).

**TABLE 2:** Performance of gray matter models.

Tasks	*r*	*P-value*	MAE
SOC	0.22 ± 0.02	*p* < 0.01	1.63 ± 0.02
SST	0.22 ± 0.02	*p* < 0.01	40.68 ± 0.47
SWM	0.38 ± 0.02	*p* < 0.01	16.96 ± 0.19
VRM	0.32 ± 0.02	*p* < 0.01	1.53 ± 0.02
DC	0.43 ± 0.01	*p* < 0.01	10.31 ± 0.12
DSYM	0.46 ± 0.01	*p* < 0.01	10.04 ± 0.11
ETSLS	0.38 ± 0.01	*p* < 0.01	4.63 ± 0.05
ETSV	0.18 ± 0.03	–	5.7 ± 0.08
LNS	0.33 ± 0.02	*p* < 0.01	2.2 ± 0.02

**FIGURE 4:**
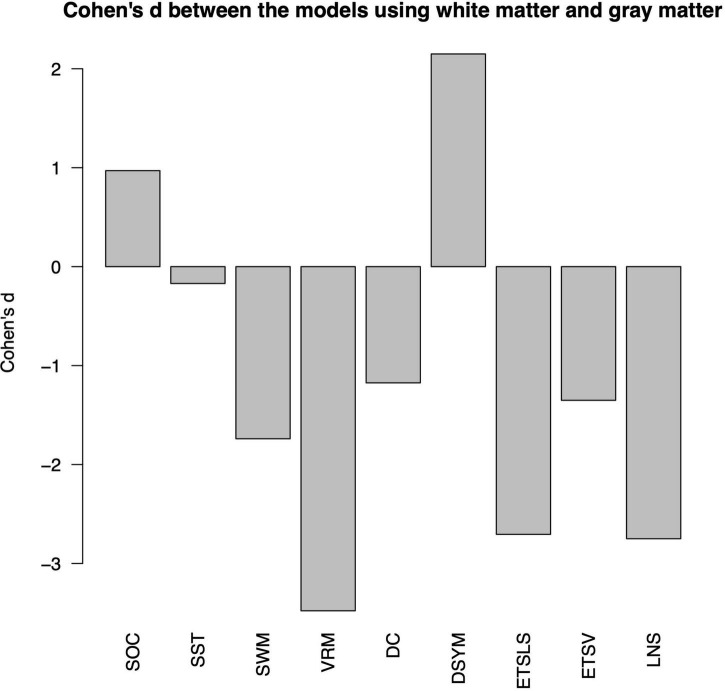
Comparisons of predictive models based on white matter and gray matter. The nine cognitive predictive models contain the Stockings of Cambridge (SOC), the stop signal task (SST), the spatial working memory (SWM), the verbal recognition memory (VRM), the digit comparison (DC), the digit symbol (DSYM), the ETS advanced vocabulary (ETSV), the ETS letter sets (ETSLS), and letter number sequencing (LNS), respectively.

Figure 4 shows the Cohen’s *d* between the performance of predictive models based on white matter and gray matter structural covariance connectivity. Most of the predictive models based on white matter structural covariance connectivity exhibit lower performance than that using gray matter connectivity. Notably, the performance of predictive models for DSYM estimation using white matter connectivity is significantly higher than that using gray matter connectivity (Cohen’s *d* = 2.15). The Cohen’s *d* > 0.8 means large effect size.

### Predictive patterns of white matter morphological connectivity

**FIGURE 5:**
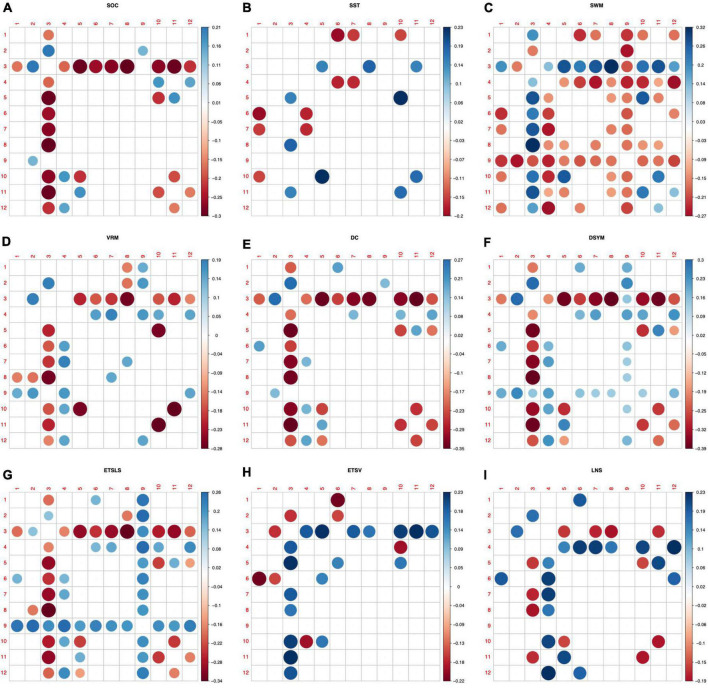
Predictive patterns of the white matter structural covariance connectivity. **(A–I)** Denote the nine cognitive tasks. The nine cognitive predictive patterns are related to the Stockings of Cambridge (SOC), the stop signal task (SST), the spatial working memory (SWM), the verbal recognition memory (VRM), the digit comparison (DC), the digit symbol (DSYM), the ETS advanced vocabulary (ETSV), the ETS letter sets (ETSLS), and letter number sequencing (LNS), respectively. Red circles denote negative correlations to cognitive scores. Blue circles denote positive correlations to cognitive scores.

Figure 5 shows the predictive patterns of white matter morphological connectivity for various cognitive tasks. The labels in the *x*-axis and *y*-axis indicate the indices of the 12 WMNs, which are discovered by a previous resting state fMRI-based study (Peer et al., 2017). Predictive patterns of the novel metrics for nine brain functions are found using feature selection (*p* < 0.05, FDR corrected). Most of the white matter structural covariance features are significantly correlated to the SWM (*p* < 0.05, FDR corrected). The sensorimotor-related white matter system (WMN-3) exhibits as the most predictive network node. The sensorimotor-related connectivity is significantly correlated to the nine brain functions (*p* < 0.05, FDR corrected). In addition, the posterior cerebellar white mater tracts (WMN-9) also exhibit as a predictive network node for SWM and the ETSLS.

### Correlations between the node strengths and cognitive functions

**FIGURE 6:**
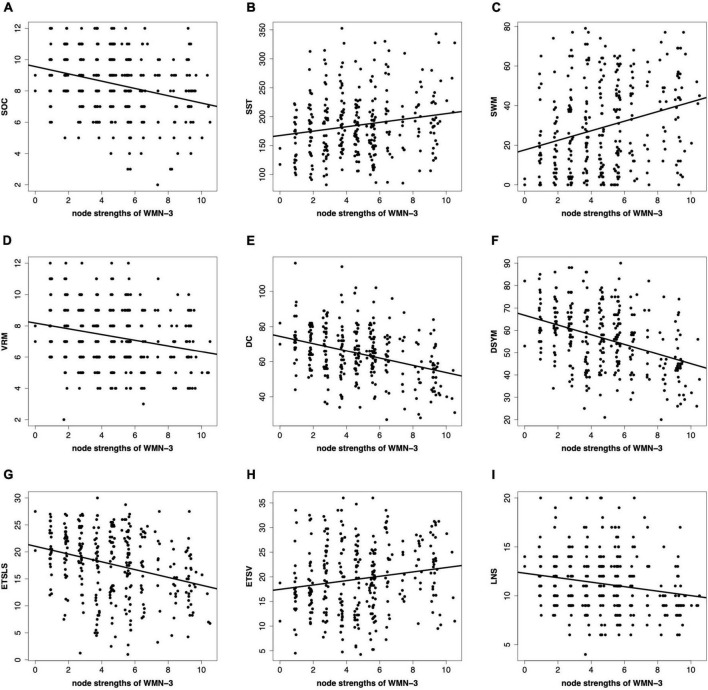
Correlations between the node strengths and cognitive scores. **(A–I)** Denote the nine cognitive tasks. The nine cognitive tasks contain the Stockings of Cambridge (SOC), the stop signal task (SST), the spatial working memory (SWM), the verbal recognition memory (VRM), the digit comparison (DC), the digit symbol (DSYM), the ETS advanced vocabulary (ETSV), the ETS letter sets (ETSLS), and letter number sequencing (LNS), respectively. The node strengths are based on the sensorimotor-related white matter network (WMN-3).

Figure 6 shows the correlations between the node strengths and cognitive functions. The node strengths of certain WMNs are significantly correlated to brain functions. In this paper, the significant correlations between the node strengths of sensorimotor-related white matter system and cognitive functions are reported in Figure 6. The sensorimotor-related white matter system is positively correlated to SST (*r* = 0.18, *p* < 0.05), SWM (*r* = 0.27, *p* < 0.05), and ETSV (*r* = 0.16, *p* < 0.05). The sensorimotor-related white matter system is negatively correlated to SOC (*r* = −0.29, *p* < 0.05), VRM (*r* = −0.23, *p* < 0.05), DC (*r* = −0.35, *p* < 0.05), DSYM (*r* = −0.39, *p* < 0.05), ETSLS (*r* = −0.29, *p* < 0.05), and LNS (*r* = −0.19, *p* < 0.05). All of the *p*-values are corrected by FDR.

### The distributions of gender, cognitive scores and age

**FIGURE 7:**
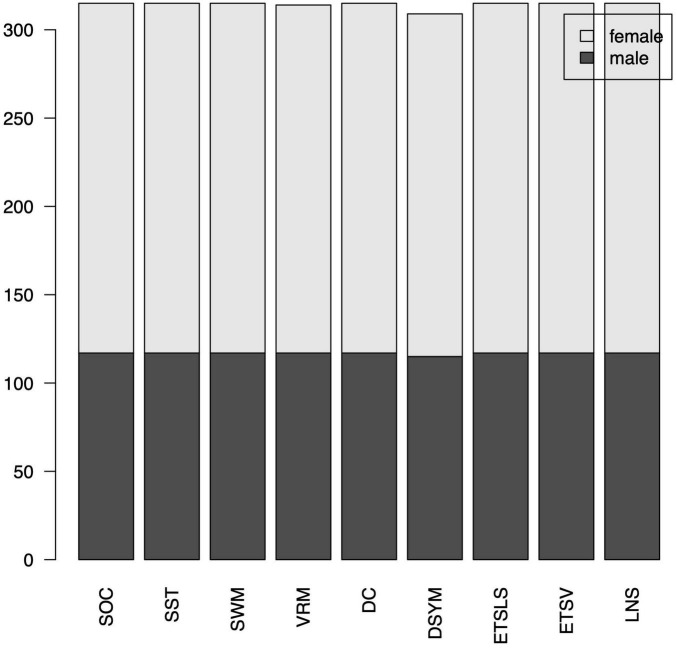
Gender distributions for the nine cognitive tasks.

**FIGURE 8:**
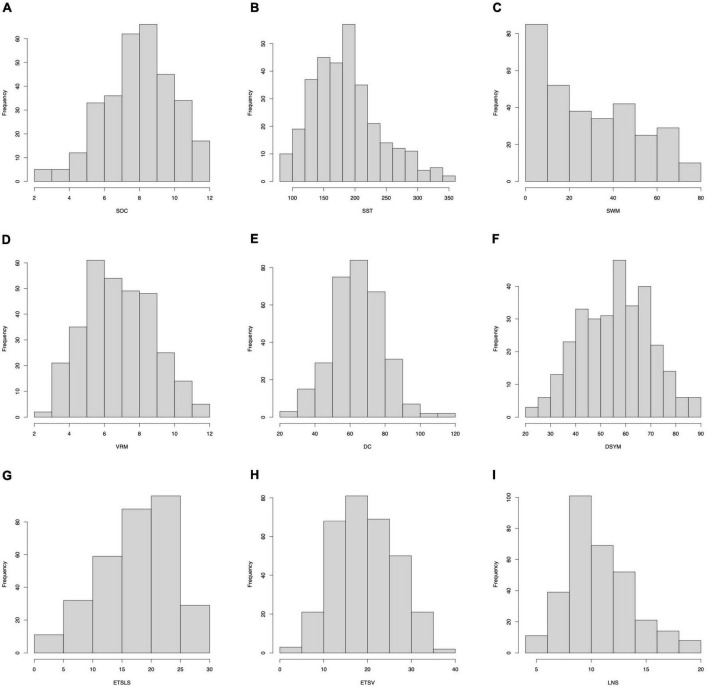
Distributions of cognitive scores. **(A–I)** Denote the nine cognitive tasks.

**FIGURE 9:**
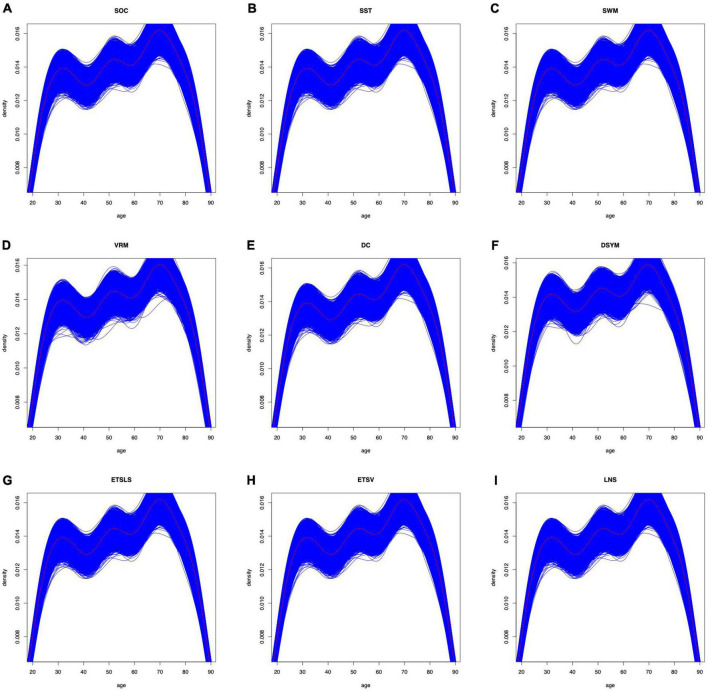
Age distributions for the training models in each task. **(A–I)** Denote the nine cognitive tasks. The blue curves fit the distributions of age in each training folds. The black curves fit the distributions of age for all samples in each task.

Of note, several cognitive scores are missing for certain subjects. The distributions of gender can be found in Figure 7. The distributions of cognitive scores can be found in Figure 8. The distributions of age can be found in Figure 9. In addition, the results of split-half analysis of age effects can be found in Tables 3, 4. Here, we only report the performance of predictive models using white matter structural covariance connectivity.

**TABLE 3:** Predicting cognitive functions of elder group using models based on younger group.

Tasks	*r*	*P-value*	MAE
SOC	0.31	*p* < 0.01	1.55
SST	0.28	*p* < 0.01	39.73
SWM	0.35	*p* < 0.01	18.64
VRM	0.19	*p* = 0.2	1.68
DC	0.38	*p* < 0.01	10.88
DSYM	0.47	*p* < 0.01	10.18
ETSLS	0.42	*p* < 0.01	4.28
ETSV	0.13	*p* = 0.1	5.6
LNS	0.23	*p* < 0.01	2.45

**TABLE 4:** Predicting cognitive functions of younger group using models based on elder group.

Tasks	*r*	*P-value*	MAE
SOC	0.28	*p* < 0.01	1.57
SST	0.24	*p* < 0.01	41.77
SWM	0.37	*p* < 0.01	17.38
VRM	0.3	*p* < 0.01	1.54
DC	0.35	*p* < 0.01	11.07
DSYM	0.39	*p* < 0.01	10.13
ETSLS	0.34	*p* < 0.01	5
ETSV	0.23	*p* < 0.01	5.79
LNS	0.22	*p* < 0.01	2.22

### Comparisons with previous morphological connectivity metrics

The wavelet-based metrics are compared with the famous KL-divergence metrics. Although both metrics are significantly correlated to age, the KL-divergence metrics fail in predicting all of the nine cognitive variables. No significant correlation is found between the KL-divergence metrics and the nine cognitive variables.

## Discussion

This paper investigated the white matter structural covariance connectivity from brain anatomical MRI, and built predictive models for the novel metrics. To achieve this goal, 3D wavelet transform was applied on the individual VBM dataset to obtain hierarchical features. The interregional connectivity was investigated using the 4D wavelet features. The SVR algorithm was then applied to build the predictive models for cognitive scores, which were well-tested using cross-validations. The predictive models achieved high performance based on the morphological connectivity. Furthermore, predictive interregional patterns were found using feature selection. The morphological connectivity was significantly correlated to cognitive scores. In summary, the novel white matter morphological features exhibited predictive power, and had potential to be neuroimaging-markers for brain disorders.

The correlation coefficient is always used to evaluate the performance of the regression models (Cui and Gong, 2018; Cohen et al., 2020). An efficient predictive model was indicated by significantly high correlation coefficients between the original scores and the predicted values. Automatic prediction of cognitive functions is a challenging task, according to the individual differences in brain activity and connectivity (Dubois and Adolphs, 2016; Scheinost et al., 2019; Pua et al., 2021). Several studies attempted to predict cognitive or behavior scores using machine learning and fMRI. The intelligence quotient (IQ), reading ability, sleep quality, inattention, impulsivity, and autistic symptoms could be predicted using machine learning (Cui et al., 2018; Cai et al., 2020; Zhou et al., 2020; Hebling Vieira et al., 2021; Wang and Li, 2021). Specially, the brain-age predictive models exhibited relatively high performance (Franke et al., 2012). The aforementioned predictive models shed lights on intelligent evaluation of human behaviors using neuroimaging-markers rather than clinical scales. However, the predictive models for various cognitive functions (i.e., SWM) remain largely unexplored. In this paper, we applied interregional white matter morphological features to predict cognitive scores for the first time. The abilities of DC and DSYM were successfully estimated using SVRs and white matter connectivity. We found that the models using white matter connectivity is better than that using gray matter connectivity for DSYM and SOC prediction. According to previous studies, the white matter was more activated than gray matter during DSYM task, suggesting that DSYM ability is more related to white matter than gray matter (Colom et al., 2010; Gawryluk et al., 2014). The SOC is a complex cognitive task that requires whole brain connectivity, which means that the white matter might play a mediation role in SOC performance improvement across childhood (Kipping et al., 2018). The performance of our predictive models was comparable to previous models. Additional results suggested that the wavelet-based metrics outperformed the KL-divergence metrics in predictive models for cognitive tasks using white matter connectivity. Notably, the predictive models were well-validated using 1,000 times of 10-fold CVs. The proposed method with high performance and reliability might outperform the conventional fMRI-based predictive models, which were quite time-consuming and related to physiological artifacts. Moreover, the predictive patterns were found using feature selection, and might open a new way to investigate the brain cognitive abilities based on white matters.

The predictive patterns of cognitive scores were discovered to investigate the brain functions. Previous studies found that the working memory network, the attentional network, and the default mode network were related to several cognitive functions (Yang et al., 2013; Rosenberg et al., 2016; Zhang et al., 2020). Most of current predictive models for brain cognitive functions were focused on the gray matter, this study provided the first evidences that brain functions could be predicted using white matter structural covariance connectivity. Specially, the sensorimotor-related white matter system (WMN-3) and posterior cerebellar white mater tracts (WMN-9) were significantly correlated to nine cognitive scores, suggesting the predictive power of the two WMNs (Peer et al., 2017). The sensorimotor and cerebellar regions of the white matter might play important roles in SWM and reading ability (Cui et al., 2018). Most of the node strengths of the WMN-3 were significantly negatively correlated to cognitive scores, implying the functional segregation in white matter with increasing abilities of cognitive functions (Fukushima et al., 2018). The predictive patterns suggested that machine learning based on white matter morphological features might be an efficient way to evaluate cognitive functions.

The biological meanings of the interregional morphological connectivity remain unclear. Nevertheless, significant correlations were found between the interregional features and cognitive scores, implying the potential biological meanings of the proposed metrics. A previous study suggested that we could explain the interregional features based on the axon tension theory (Van Essen, 1997; Kong et al., 2014), which assumed that the structurally linked brain regions were connected by a mechanical force. Therefore, the two linked brain regions exhibited similar radiomic features. Another previous study found that the interregional morphological connectivity exhibited discriminative powers for attention deficit hyperactivity disorder (ADHD) identification (Wang et al., 2018a). The interregional features were significantly related to individual inattention and impulsivity (Wang et al., 2018a), and could predict the clinical severity of autism spectrum disorder (ASD) (Sato et al., 2013). One of our previous study also found the voxel-wise morphological features exhibited high reliability and could represent individual differences (Wang et al., 2018b). The above evidences suggested that the morphological connectivity had potential discriminative and predictive powers in machine learning tasks. Although lack of interpretation of its biological meanings, the interregional white matter morphological connectivity still had potential to be a novel neural-metric for brain connectome.

In addition, we analyzed the age effects on the predictive models. First, the 10-fold cross-validation procedures were repeated 1,000 times for each predictive model to avoid random sampling. Second, the distributions of age variable were plotted in Figure 9. We found that the curves of distributions for age variable were similar to the original ones. Third, the subjects were reassigned into two groups: younger group (age < mean age) and elder group (age > mean age). We trained the nine predictive models based on the younger group and tested the models using the elder group, and obtained desired performance. We then reversed the training and testing samples, and also obtained significant performance. Forth, we found that none of the nine cognitive variables was correlated to age. We then combined the structural covariance connectivity and age as features for the predictive models, and no significant improvement was found for the model performance. In summary, age effects might play less important roles in this study, according to current evidences.

This study was performed with several advantages. One advantage was predicting cognitive scores based on interregional white matter morphological features. The conventional regional morphological measures could only represent the local information of brain regions. Our method could provide additional brain topology information for the white matters. Furthermore, the proposed brain network features were significantly related to cognitive scores, suggesting the potential biological basis of the morphological connectivity. Therefore, the interregional morphological connectivity was informative. Another advantage was validating the variability of the proposed metrics using the machine learning. The performance of the predictive models was comparable to previous methods, implying the potential clinical applications of the proposed metrics. The predictive models could open a new perspective for brain disorders and healthy aging.

There were several limitations that should be addressed in future study. One limitation was the explanation of the proposed morphological metrics for white matter. Although the interregional features were significantly correlated to the cognitive scores, additional cognitive tasks and different imaging modalities (i.e., functional fMRI, diffusion MRI) should be applied to explain the biological meanings of the structural covariance connectivity. Furthermore, other novel feature extraction methods for investigating the morphological connectivity should be analyzed in subsequent study. Another limitation was the morphological connectivity depended on the wavelet basis and scales. The criteria for wavelet scale selection should be addressed in future research. The third limitation was the white matter functional atlas used in this paper. Although the white matter functional networks were well-established using resting state fMRI and diffusion MRI, a novel white mater atlas derived from morphological features should be analyzed and validated in future study. We agree that individualized functional network parcellation is beneficial for behavior prediction (Kong et al., 2021). The T1-weighted MRI wavelets features can also be used for individual brain parcellations. We sought to let the potentials of wavelets transforms on brain parcellations as future directions. The fourth limitation was the machine learning procedure, various feature selection and regression algorithms should be compared in future study. The fifth limitation was the study population, which was mixed with the *apoe*-gene carriers. Increased sample size and different kinds of populations should be investigated additionally. The last limitation was the parameters of the MRI, which had impacts on the VBM measures (Streitbürger et al., 2014). Different spatial resolutions, scan sequences, head coils of MRI scan sessions should be compared in future research.

## Conclusion

This paper proposed a novel neural-metric named white matter structural covariance connectivity based on wavelet transform. The cognitive scores were estimated using the interregional morphological features. The predictive models for several cognitive functions achieved high performance based on cross-validations. The predictive patterns of interregional morphological connectivity for cognitive scores were found by machine learning. The results suggested that the interregional white matter morphological connectivity could be a potential neural-metric for brain connectome.

## Data availability statement

The datasets presented in this study can be found in online repositories. The names of the repository/repositories and accession number(s) can be found below: http://fcon_1000.projects.nitrc.org/indi/retro/dlbs.html.

## Ethics statement

The studies involving human participants were reviewed and approved by the International Neuroimaging Data-sharing Initiative (INDI) and the University of Texas at Dallas. Written informed consent was not needed in accordance with the local legislation and institutional requirements.

## Author contributions

X-HW and LL contributed to the conception and design of the study. X-HW, BZ, and LL wrote the first draft of the manuscript. X-HW and BZ performed the statistical analysis. All authors contributed to the manuscript revision, read, and approved the submitted version.

## Footnotes

1
http://fcon_1000.projects.nitrc.org/indi/retro/dlbs.html

2
https://fsl.fmrib.ox.ac.uk/fsl/fslwiki/FSLVBM

3
https://github.com/xunhengwang/MCWT

4
https://cran.r-project.org/web/packages/e1071/index.html

5
www.nitrc.org/projects/bct

6
https://cran.r-project.org/web/packages/RVAideMemoire/
